# Bioprotective Role of Yeasts

**DOI:** 10.3390/microorganisms3040588

**Published:** 2015-10-10

**Authors:** Serena Muccilli, Cristina Restuccia

**Affiliations:** 1 Consiglio per la Ricerca in Agricoltura e L’analisi dell’Economia Agraria-Centro di Ricerca per l’Agrumicoltura e le Colture Mediterranee, Corso Savoia 190, 95024 Acireale, CT, Italy; E-Mail: serenamuccilli@hotmail.com; 2 Di3A-Dipatimento di Agricoltura, Alimentazione e Ambiente, University of Catania, via Santa Sofia 98, 95123 Catania, Italy

**Keywords:** biocontrol, diseases, food industry, killer toxins, nutrient competition, postharvest, *Wickerhamomyces anomalus*, yeast species

## Abstract

The yeasts constitute a large group of microorganisms characterized by the ability to grow and survive in different and stressful conditions and then to colonize a wide range of environmental and human ecosystems. The competitive traits against other microorganisms have attracted increasing attention from scientists, who proposed their successful application as bioprotective agents in the agricultural, food and medical sectors. These antagonistic activities rely on the competition for nutrients, production and tolerance of high concentrations of ethanol, as well as the synthesis of a large class of antimicrobial compounds, known as killer toxins, which showed clearly a large spectrum of activity against food spoilage microorganisms, but also against plant, animal and human pathogens. This review describes the antimicrobial mechanisms involved in the antagonistic activity, their applications in the processed and unprocessed food sectors, as well as the future perspectives in the development of new bio-drugs, which may overcome the limitations connected to conventional antimicrobial and drug resistance.

## 1. Introduction: Yeast Potential in Assuring Food Safety

Chemical food preservatives are commonly used to extend the shelf life and to improve the safety of food by inhibiting the growth of spoilage and pathogenic bacteria. However, increasing consumer fears about their potential toxicity and antimicrobial-resistant pathogens present in food, which constitute a direct risk to public health, have prompted research into alternative and safer methods of food preservation, of which biopreservation has been perceived as a potential substitute [1,2].

Biopreservation or biocontrol refers to the use of natural or controlled microorganisms, or their antimicrobial products, to extend the shelf life and to enhance the safety of food [3], and it can be achieved by either (1) the addition of antimicrobial metabolites without the producing strain, (2) the addition of a culture producing antimicrobial metabolites that does not influence food quality or (3) the application of pro-technological microorganisms harboring protective effects. A number of microorganisms and other biological agents have been regarded to be crucial in the biopreservation of food, indirectly (by changing pH or osmotic pressure) or directly (by producing toxic compounds, antimicrobial components, enzymes, antibiotics, *etc*.).

Although the most intensive studies and practical applications of microbial antagonisms have focused on lactic acid bacteria (LAB) [4], considerable research has been aimed in the past two decades at investigating the use of naturally-occurring yeast for inhibiting the growth of food-borne bacteria and for managing postharvest diseases on a variety of fruits and vegetables with various mechanisms [5,6].

All yeasts are eukaryotic microorganisms, which are most commonly defined as unicellular fungi, although unicellular growth occurs within several fungal taxonomic orders and many types of yeast can grow by forming pseudo-hyphae.

In nature, yeast species are found mainly in association with plants or animals, but are also present in soil and aquatic environments [7]. They colonize an extremely wide range of ecosystems, both natural and in connection with human activities, mainly for their ability to grow and survive in different and stressful environments [8]. Few other microbial organisms match yeast in terms of historical, economic and scientific significance, as the spontaneous fermentation of wine, beer and cereal doughs is one of the oldest biopreservation technologies, empirically used since ancient times. Among the potential microbial antagonists, yeasts have been extensively studied because they possess many features that make them suitable as biocontrol agents [6,9]. Many yeast species have simple nutritional requirements, and they are able to colonize dry surfaces for long periods of time and can grow rapidly on inexpensive substrates in bioreactors, characteristics that are particularly relevant in the selection of biocontrol agents [10]; however, understanding the ecological fitness of the potential yeast biocontrol agents and developing strategies to enhance their stress tolerance are essential to their efficacy and commercial application [11]. Moreover, they do not produce allergenic spores or mycotoxins, as many mycelial fungi or antibiotics that might be produced by bacterial antagonists [12,13].

## 2. Antagonistic Mechanisms of Yeast

Antagonistic characteristics of yeast have been attributed mainly to: (1) competition for nutrients; (2) pH changes in the medium as a result of growth-coupled ion exchange or organic acid production; (3) tolerance to high concentrations of ethanol [14]; and (4) the secretion and release of antimicrobial compounds, such as killer toxins or “mycocins” [15,16,17,18].

According to Do Carmo-Sousa [19], competition for nutrients is probably the most important factor in yeast ecology. The competition for nutrients is considered to be a primary mode of action against postharvest fungal pathogens, especially in fruits [20,21]. Yeast promptly depletes glucose, fructose or sucrose, preventing the growth of undesirable microorganisms, as already extensively exploited in food and beverage fermentation for the species *Saccharomyces cerevisiae*. Moreover, the sugar competition was demonstrated in the antagonist pink yeast *Sporobolomyces roseus* against *Botrytis cinerea* [22] and in the *Pichia guilliermondii* species against *Ceratocystis paradoxa* [23]. The competition for nitrogenous compounds, especially in the carbon-rich environment of fruit wounds, was determined for *Candida sake* [24] and *Candida guilliermondii* [25] against *Penicillium expansum*. Iron is essential for microbial growth and also for pathogenesis; the production, release and uptake of iron-scavenging molecules, called siderophores, is a major microbial mechanism for iron acquisition [26], and numerous examples of siderophore-mediated interspecies competition have been described. Iron sequestration by yeast has been exploited in novel systems for biological control of postharvest pathogens sensitive to iron deprivation [27,28]. This primary competition strategy was demonstrated in the species *Metschnikowia pulcherrima*, which produces the red pigment pulcherrimin in the presence of iron, indicating the uptake of ferric ions from the surrounding substrate, against the fungal pathogens *B. cinerea*, *Alternaria alternata* and *P. expansum* [29,30].

Yeast killer toxins, also named mycocins, were initially defined as extracellular proteins, glycoproteins or glycolipids that disrupt the cell membrane function in susceptible yeast bearing receptors for the compound [16,31,32], whose activity is directed primarily against yeast closely related to the producer strain, which has a protective factor. The first mycocins were identified in association with *S. cerevisiae* in the brewing industry [33]. Several others have since been isolated, frequently where yeast populations occur in high density and in highly competitive conditions, as for example fermented olive brine [34,35,36] and fermenting grape must [37]. Killer toxin production has been demonstrated among many yeast genera, including *Saccharomyces*, *Candida*, *Cryptococcus*, *Debaryomyces*, *Kluyveromyces*, *Pichia*, *Torulopsis*, *Williopsis* and *Zygosaccharomyces* [38,39,40,41,42,43,44]. Genetic and molecular studies have shown that the killer toxin feature may be carried out on extra-chromosomal elements, such as double-stranded RNA viruses [45,46], on double-stranded linear DNA [42,47,48] or on a chromosome [42,49,50].

The well-known mechanisms of the killer toxin against other fungi are the inhibition of β-glucan synthesis or hydrolysis of β-glucan in the cell wall of sensitive strains [44,51,52,53,54], the interruption of cell division by blocking the DNA synthesis [52,55,56], the cleavage of tRNA [57], the blocking of calcium uptake [55,58] and the ion leakage caused by the formation of channels on the cytoplasmic membrane [18,59,60].

Unlike yeast-against-yeast antagonism, the antibacterial properties of yeast are much less documented. The first positive indications of the antagonistic activity of yeast were published in the early 20th century [61] from Hayduck, who reported a volatile thermolabile toxic extract from yeast, probably an amine, which inhibits the growth of *Escherichia coli* and *Staphylococci* [62]. Fatichenti *et al.* [63] showed that the antibacterial activity of *Debaryomyces hansenii* against *Clostridium tyrobutyricum* and *Clostridium butyricum* was related to its ability to produce both extracellular and intracellular antimicrobial compounds. Antibacterial activity was also detected in *Kloeckera apiculata* and *Kluyveromyces thermotolerans*, secreting substances that inhibited the growth of beer-spoilage bacteria [64]. Polonelli and Morace [65] also reported on a killer phenomenon directed against a wide range of unrelated microorganisms, among others, bacteria. Dieuleveux *et al.* [66] subsequently described the inhibition of *Listeria* by a strain of *Geotrichum candidum* isolated from a French red smear cheese able to synthetize d-3-phenyllacticand d-3-indollactic acids. Furthermore, Cavalero and Cooper [67] demonstrated that *Candida bombicola* produces extracellular glycolipids, called sophorosides, which have been proven to have antibacterial activity mainly against Gram-positive bacteria [68]. Having tested about 400 yeast isolates, mainly from dairy sources, Goerges *et al.* [69] reported a strain of *Candida intermedia* able to reduce viable *L. monocytogenes* counts by four log units and a strain of *Kluyveromyces marxianus* able to suppress the pathogen growth by three log units. The same author isolated a strain of *Pichia norvegensis* able to reduce *L. monocytogenes* counts by seven log cycles [70]. Moreover, Hatoum *et al.* [71] characterized anti-listerial hydrophobic peptides, extracted from four dairy yeast cultures identified as *D. hansenii*, *Pichia fermentans*, *Candida tropicalis* and *Wickerhamomyces anomalus*, which induced leakage in bacterial cells and ultimately caused bacterial lysis. More recently, Chen and co-workers [72] isolated two strains of *Kluyveromyces marxianus* producing mycocins from Koumiss in Inner Mongolia and demonstrated that the two crude extracts were effective at preventing *Escherichia coli* disease in mice. Finally, a very recent study, aimed at evaluating some probiotic properties of the *P. pastoris* strain X-33 wild-type, demonstrated the growth inhibition of *Salmonella typhimurium* *in vitro* and the reduction of bacterial adhesion to the human colorectal cancer HCT-116 cells [73].

## 3. Applications of Antagonistic Activities of Yeast in Foods

### 3.1. Processed Food and Beverages

It is widely recognized that the overall product quality in industries, such as winemaking, sausage and dairy production, baking, *etc.*, is mainly correlated with the development of spoilage microorganisms [74,75,76]. In the past few decades, several studies were focused on the application of antagonistic yeast starter cultures in various food and beverage processes for improving their safety and sensory qualities, respectively, by the inhibition of pathogenic and spoilage organisms (Table 1).

Although *S. cerevisiae* wine starter cultures are normally able to dominate native yeast in the grape must during fermentation [77], most studies have validated the use of killer yeast as starter cultures to prevent the growth of spoilage yeast and bacteria in wine fermentations [78,79,80,81,82,83]. In the production of sparkling wine, Todd *et al.* [84] studied the behavior of two sensitive strains of *S. cerevisiae* in the presence of a mixture of two K2 killer toxins, coming to the conclusion that this interaction accelerates the yeast autolysis and, as a consequence, the release of proteins that affects the end product quality. *S. cerevisiae* strains that produce K2 and Klus toxins were found to be effective for preventing the growth of spoilage yeast strains [79,85,86]; however, numerous non-*Saccharomyces* yeast species present on the surface of grapes are insensitive to the *S. cerevisiae* killer toxins and they are able to inhibit the growth of spoilage yeast. The killer toxin secreted by *Tetrapisispora phaffii*, named KpKt by Comitini and Ciani [87], has been identified as a β-glucanase with extensive anti-*Hanseniaspora*/-*Kloeckera* activity under winemaking conditions, by inducing ultrastructural modifications in the cell wall of sensitive strains, with a high specific cytocidal activity, a selective action towards target yeast cells [88] and, thus, potentially useful for the wine industry. Other studies of Comitini and co-workers [81,89] showed that killer toxins secreted by *W. anomalus* (Pikt) and *Kluyveromyces wickerhamii* (Kwkt) are active against *Dekkera* and *Brettanomyces* spoilage yeast species that cause unpleasant odors in wine during fermentation, ageing and storage. The killer activity of autochthonous *W. anomalus* from the northwest region of Argentina was demonstrated against *Brettanomyces bruxellensis*, *Dekkera anomala*, *Pichia membranifaciens* and *Meyerozyma guilliermondii* [90]. Numerous studies have confirmed remarkable inhibitory properties of the killer toxins of *W. anomalus* and *Williopsis mrakii* [51,91,92,93] against a wide range of pathogenic and spoilage fungi. Two killer toxins, CpKT1 and CpKT2, from the wine-isolated yeast *Candida pyralidae* exhibited killer activity against several *B. bruxellensis* strains, especially in grape juice, but did not inhibit the commercial *S. cerevisiae* tester strain [94]. Similarly, the killer activity of *Ustilago maydis* producing a KP6-related toxin was proven to be effective against *B. bruxellensis*, while *S. cerevisiae* was fully resistant to its killer activity [95]. The antagonistic properties of yeast can also influence the interactions of wine yeast and malolactic bacteria, mainly *Oenococcus oeni*, stimulating or preventing the progress of malolactic fermentation that improves wine stability and quality [96]. With this aim, screening against undesirable lactic acid bacteria (LAB) showed that killer toxins produced by *S. cerevisiae* and *W. anomalus* were able to inhibit the growth of *Lactobacillus hilgardii*, as well as its histamine production [90].

Killer toxin-producing yeast strains have also been proposed for multiple applications in the production of beer [97] and sake [98], although this phenotype has been broadly found in salted fermented food, such as fermented olive [35,36,99,100,101,102]. Salt, in fact, is necessary to reveal the killer phenotype of some yeast species [97] and may broaden the activity spectra of a killer yeast against target strains [103]; as killer toxins induce the formation of ion-permeable channels in lipid bilayer membranes, causing a disruption of the ionic equilibrium across the plasma membrane, as reported by Kagan [104], the incidence of salt increases the mortality of the intoxicated cells [99]. The killer character has also been exploited in bread production by the use of hybridized wild killer yeast with an industrial strain of *S. cerevisiae* [105]. Pérez-Nevado *et al.* [106] isolated high percentages of killer yeast on Jamón de Huelva and Dehesa de Extremadura SDO dry-cure hams, while Virgili *et al.* [107] successfully used killer yeast strains isolated from the surface of Italian typical dry-cured hams, to control the growth of a toxigenic strain of *Penicillium nordicum* and to inhibit the ochratoxin A (OTA) biosynthesis.

In the dairy sector, the use of killer starter yeast strains to prevent spoilage in cheese [63,108,109], yogurt [93,110,111,112,113] and other foods [114] has also drawn considerable attention.

However, one of the main factors that should be taken into consideration for the application of killer toxins in food processes is the pH and the temperature range at which the activity is high, as it can limit their effectiveness, especially in food fermentations. Although there is large variation in the optimal pH and temperature conditions for various toxins, they are generally active between pH values of 4.0 and 5.4 and at temperatures below 30 °C [41,115,116]. However, Hara *et al.* [117] reported an effective killer action of a hybrid *S. cerevisiae* culture at pH values between 3.0 and 4.5 and at temperatures between 15 and 35 °C, and Ciani and Fatichenti [118] reported an extensive anti-*Hanseniaspora* activity of *Tetrapisispora phaffii* (formerly *Kluyveromyces phaffii*) DBVPG (Industrial Yeast Collection of the University of Perugia) 6076 in the pH range between 3 and 5 and at temperatures lower than 40 °C, making them both suitable for wine making. Another example of killer toxin that is active in a broader range of pH values and temperatures than what is described for other zymocins is CnKT (Candida nodaensis Killer Toxin) [119], from the extreme halotolerant yeast *Candida nodaensis*, as it proved to be active between pH 2.6 and 6.0 and at temperatures ranging from 18 to 30 °C; moreover, its activity was stimulated by sodium ions, making CnKT a promising candidate for several biotechnological applications, e.g., in the preservation of high-salt food products from spoilage by other yeasts.

### 3.2. Unprocessed Foods

In addition to their role in the production of processed foods and beverages, yeast antagonize spoilage or toxin-producing microorganisms in unprocessed foods by several mechanisms (Table 2). The most promising strategy to achieve this objective seems to be the use of specific yeast strains exhibiting such inhibitory features, selected among the epiphytic microbial community of fruits and vegetables and then phenotypically adapted to this niche. The advantage of being part of the natural microbial community already established on the target product may facilitate their colonization and survival on produce when applied in appropriate numbers.

Several yeast species naturally occurring on the surface of fruits and vegetables have been widely studied for the control of postharvest diseases [120], to reduce the chemical residue on fresh products and to bypass the developing resistances to widely-used synthetic fungicides [121]; for their efficacy, stability, safety and ease of application, yeast-based biocontrol products are already available on the market and are registered on several commodities against rots caused by genera *Penicillium*, *Aspergillus*, *Botrytis* and *Rhizopus* [6].

The biocontrol abilities of *S. cerevisiae* and *W. anomalus* strains have been recently proven to be correlated with killer phenotype [122,123], while in other yeast species, the antagonistic activity has been mainly attributed to competition for nutrients and space, production of hydrolytic enzymes or volatile organic compounds (VOCs). In particular, the competition for iron was reported to play a significant role in biocontrol interactions of *M. pulcherrima* [29]; yeast strains belonging to this species are effective against postharvest decay of apple, table grape, grapefruit, cherry tomato, sweet cherries and peach [30,124,125,126,127,128,129]. The antagonistic activity of *Aureobasidium pullulans* mainly includes nutrient competition [130] and production of glucanase, chitinase, protease and extracellular proteases [131,132]. The ability to rapidly colonize the fruit tissues, competing with the pathogens *B. cinerea* and *P. expansum* for nutrients, has been recognized as the main mechanism responsible for the antagonistic efficacy on pear fruits [133].

With regards to VOCs, the ethyl acetate produced by *W. anomalus* has been shown to have antifungal activity during airtight storage of grain [134,135], while more recently, Hua *et al.* [136] stated that its biocontrol ability can be attributed to the production of 2-phenylethanol, which affects spore germination, growth, toxin production and gene expression in *Aspergillus flavus*. A similar mechanism based on VOC production has been identified by Fiori and co-workers [137] for two non-fermenting (*Cyberlindnera jadinii* and *Candida friedrichii*) and two low-fermenting (*Candida intermedia* and *Lachancea thermotolerans*) yeast strains against the pathogenic fungus and OTA-producer *Aspergillus carbonarius*.

Yeast strains isolated from sugar cane and maize rhizosphere, leaves and stalks, identified as *Torulaspora globosa* and *C. intermedia*, were able to inhibit the growth of phytopathogenic molds *Colletotrichum sublineolum* and *Colletotrichum graminicola*, both causal agents of the anthracnose disease in, respectively, sorghum and maize, with the first species also exhibiting killer activity [138].

The antagonistic mechanism based on hydrolytic enzyme production, together with competition for nitrogen and carbon sources and induction of host resistance, was demonstrated for *Meyerozyma guilliermondii* strain M8 against *B. cinerea* on apples [139]. Lytic enzymes were similarly produced by epiphytic isolates of *Rhodotorula mucilaginosa* and *Candida famata* in controlling postharvest anthracnose in papaya fruit caused by *Colletotrichum gloeosporioides* [140].

In the last few years, the *W. anomalus* species, which is frequently associated with food and feed products, has been extensively studied for the widely intergeneric killing spectrum of produced toxins against postharvest spoilage molds. The antimycotic properties of *W. anomalus* against grain storage fungi were originally described by Björnberg and Schnürer in 1993 [141]. Later, Jijakli and Lepoivre [142] proposed that the suppression of *B. cinerea* by *W. anomalus* is partly due to the activity of an exo-β-1,3-glucanase, while Masih *et al.* [143] showed that *B. cinerea* displays emptied hyphae when in contact with *W. anomalus* yeast cells. More recently, similar investigations by Mohamed and Saad [144] have shown by scanning electron microscopy the antagonistic effects of *W. anomalus* cells interacting with the fungus *Botryodiplodia theobromae*. The analysis showed pitting and disruption on hyphal surfaces that were totally penetrated and killed. Druvefors and Schnürer [145] found that *W. anomalus* was the best yeast among 60 different tested yeast species with regards to the inhibition of *Penicillium roqueforti* growth in test tube versions of airtight grain silos; in addition, its inoculation to cereal feed grain improved feed hygiene by reducing molds and *Enterobacteriaceae* and enhanced the nutritional value by increasing the protein content and reducing the concentration of the antinutritional compound phytate [146].

Exo-β-1,3 glucanases have been shown to contribute to the mechanism of action of the antagonistic yeast *W. anomalus* (strain K) against *B. cinerea* and *P. expansum* on apples [147,148], *B. cinerea* on grapes [30], *Penicillium digitatum* on “Tarocco” and “Valencia” oranges [123,149] and *C. gloeosporioides* on papayas [150].

**Table 1:** Yeast strains and antagonistic mechanisms in processed food and beverage applications.

Species	Yeast Strain	Mechanism	Application	References
*C. pyralidae*	IWBT Y1140, IWBT Y1057	n.s.	In grape juice agst. *B. bruxellensis*	[94]
*D. hansenii*	CYC (Complutense Yeast Collection) 1021	n.s.	In olive fermentation agst. *C. boidinii*, *S. exiguous* and *K. lactis*	[99]
*D. hansenii*	B9010	n.s.	In yoghurt and on cheese at non-refrigerated agst. *Aspergillus*, *Byssochlamys*, *Eurotium*	[110]
*D. hansenii, D. marasmus, C. zeylanoides, C. famata, H. burtonii*	n.s.	n.s.	In ham agst. *P. nordicum* growth and OTA production	[106,107]
*Filobasidium floriforme*	NRRL Y7454	sugar competition	In apple agst. *B. cinerea*	[22]
*Kluyveromyces wickerhamii (Kwkt)*	n.s.	n.s.	In wine agst. *Dekkera* and *Brettanomyces*	[81,89]
*P. membranaefaciens*	CYC 1106, CYC 1108	n.s.	In table olive fermentation agst. *C. boidinii*	[99]
*S. cerevisiae*	n.s.	K2 and Klus toxins	In winemaking agst. spoilage yeast strains	[79,84,85]
*S. cerevisiae*	CF-K*115	dsRNA	In sake fermentation	[98]
*S. cerevisiae*	CYC 1115	n.s.	In table olive fermentation agst. *C. boidinii*	[99]
*S. cerevisiae*	Itati K^+^	n.s.	Transfer of killer particles to the industrial strain for the bakery industry	[105]
*S. cerevisiae*	Cf8, M12	n.s.	In winemaking agst. *Brettanomyces bruxellensis*, *Dekkera anomala*, *Pichia membranifaciens*	[122]
*Tetrapisispora phaffii*	DBVPG 6706	β-glucanase	In winemaking agst. *Hanseniaspora*/*Kloeckera*	[87]
*Torulaspora globosa*	1S100, 1S111, 1S112, 2S01, 2S04, 2F58	Killer toxin	In sorghum and maize agst. *Colletotrichum sublineolum* and *Colletotrichum graminicola*	[138]
*Ustilago maydis*	CYC 1410	KP6 toxin, ion channel, possibly causing the leakage of K^+^ or NH4^+^ from cells	In grape juice agst. *B. bruxellensis* strains	[95]
*W. anomalus*	CYC 1027	n.s.	In table olive fermentation agst. *C. boidinii*	[99]
*W. anomalus*	Cf20	n.s.	In winemaking agst. *Brettanomyces bruxellensis*, *Dekkera anomala*, *Pichia membranifaciens* and *Meyerozyma guilliermondii*	[90]
*W. anomalus*	(Pikt)	n.s.	In winemaking agst. *Dekkera* and *Brettanomyces*	[81]
*Williopsis mrakii*	LKB (Laboratory of Kodama Brewery) 169 = NCYC (National Collection of Yeast Culture) 251	n.s.	In yogurt and maize silage agst. *Candida krusei* D1241 and *Saccharomyces cerevisiae* D1247	[93]
*W. saturnus* var*. saturnus*	CBS254	Competition for space and killer toxin	In cheese biopreservation agst. *S. cerevisiae* and *K. marxianus* In yoghurt agst. *C. kefir*, *K. marxianus*, *S. cerevisiae*, *S. bayanus*, *Byssochlamys*, *Eurotium* and *Penicillium*	[111,112,113]

n.s., not specified.

**Table 2:** Yeast strains, mechanism of action and applications against postharvest pathogenic molds on different commodities.

Species	Yeast Strain	Mechanism	Application	References
*Aureobasidium pullulans*	PI1	n.s.	On grape berries agst. *B. cinerea*	[30]
*A. pullulans*	PL5	β-1,3-glucanase, exochitinase, endo-chitinase and competition for nutrients and space	On plums and peaches agst. *M. laxa* On apples agst. *B. cinerea and P. expansum* On stone fruit agst. *M. laxa* On pome fruits agst. *B. cinerea* and *P. expansum*	[127,131]
*A. pullulans*	LS-30	Competition for nutrients; extracellular exochitinase (*N*-acetyl-β-d-glucosaminidase (Nagase)) and β-1-3-glucanase	On table grapes agst. *B. cinerea, P. expansum, Rhizopus stolonifer* and *A. niger* On apple fruit agst. *B. cinerea* and *P. expansum*	[130]
*A. pullulans*	L47	Competition for nutrients	On strawberries grown under plastic tunnels agst. *B. cinerea* and *R. stolonifer*; on apple agst. *B. cinerea* and *P. expansum*	[132,133]
*C. intermedia*	235	VOCs	On grape berries agst. *A. carbonarius* and ochratoxin A (OTA) contamination in wine and grape juice	[137]
*C. oleophila*	L66	Competition for nutrients	On strawberries grown under plastic tunnels agst. *B. cinerea* and *R. stolonifer*	[133]
*Candida famata*	n.s.	Lytic enzyme	On papaya agst. *Colletotrichum gloeosporioides*	[141]
*Candida friedrichii*	778	n.s.	On grape berries agst. *A. carbonarius* and ochratoxin A (OTA) contamination in wine and grape juice	[137]
*Candida guilliermondii*	3C-1b, 1F	Competition for nitrogenous compounds	On apple against *Penicillium expansum*	[25]
*Candida intermedia*	2S02, 2S03	n.s.	In sorghum and maize agst. *Colletotrichum sublineolum* and *Colletotrichum graminicola*	[138]
*Candida sake*	n.s.	Competition for nitrogenous compounds	On pear against *Penicillium expansum*	[24]
*Candida vanderwaltii*	L60	n.s.	On strawberries grown under plastic tunnels agst. *B. cinerea* and *R. stolonifer*	[132]
*Cryptococcus laurentii*	LS28	n.s.	On apples for integrated control of *P. expansum* and patulin	[128]
*Cryptococcus humicola*	NRRL Y1266	Sugar competition	In apple agst. *B. cinerea*	[22]
*Cyberlindnera jadinii*	273	n.s.	On grape berries agst. *A. carbonarius* and ochratoxin A (OTA) contamination in wine and grape juice	[137]
*Lachancea thermotolerans*	751	n.s.	On grape berries agst. *A. carbonarius* and ochratoxin A (OTA) contamination in wine and grape juice	[137]
*M. pulcherrima*	MPR3	n.s.	on grape berriesagst. *B. cinerea*	[30]
*M. pulcherrima*	ST1-D10, ST2-A10, ST3-E1, ST3-E13, T4-A2, T5-A2, FMB-24H-2, FMB-140H-7A	n.s.	On apple agst. *P. expansum*	[125]
*M. pulcherrima*	GS37, GS88, GA102, BIO126	Competition for nutrient and space	On apples agst. *B. cinerea* and *P. expansum*	[126]
*Metschnikowia spp.*	LS15	Competition for nutrient and space	On table grape agst. *B. cinerea*	[124]
*M. pulcherrima*	MACH1	Iron competition	On apples agst. *B. cinerea*, *Alternaria alternata* and *P. expansum*	[29]
*M. pulcherrima*	Disva 267	n.s.	On sweet cherries agst. *Monilinia laxa*	[129]
*Meyerozyma guilliermondii*	443	β-1,3-glucanase	On papaya agst. *C. gloeosporioides*	[150]
*Pichia guilliermondii*	Pichia	Sugar competition	On pineapple agst. *Ceratocystis paradoxa*	[23]
*Pichia guilliermondii*	M8	β-1,3-glucanase and chitinase	On apples agst. *Botrytis cinerea*	[139]
*Rhodosporidium kratochvilovae*	LS11	n.s.	On apples agst. *P. expansum* and patulin	[121]
*Rhodosporidium toruloides*	NRRL Y1091	Sugar competition	In apple agst. *B. cinerea*	[22]
*Rhodotorula mucilaginosa*	n.s.	Lytic enzyme	On papaya agst. *Colletotrichum gloeosporioides*	[140]
*Sporobolomyces roseus*	FS-43-238	Sugar competition	In apple agst. *B. cinerea*	[22]
*W. anomalus*	Disva 2	n.s.	On sweet cherries agst. *Monilinia laxa*	[129]
*W. anomalus*	J121	Ethyl acetate and ethanol derived from glycolysis	In airtight storage of wheat agst. *Penicillium roqueforti* and *Enterobacteriaceae*	[136,137,138,139,140,141,142,143,144,145,146,147]
*W. anomalus*	WRL-076	VOCs (2-phenylethanol)	In tree nuts agst. *Aspergillus flavus*	[136]
*W. anomalus*	Strain K	exo-β-1,3-glucanase	On apples agst. *B. cinerea*	[142,147,148]
*W. anomalus*	strain FY-102	n.s.	On grape vine agst. *B. cinerea*	[143]
*W. anomalus*	Moh 93, Moh 104	n.s.	On guava (Psidium guajava L) agst. *Botryodiplodia theobromae*	[144]
*W. anomalus*	422	β-1,3-glucanase	On papaya agst. *C. gloeosporioides*	[150]
*W. anomalus*	BS91, BS92, BCA15	β-glucanase	On orange agst. *Penicillium digitatum* On grape berries agst. *B. cinerea*	[30,123,149]

Notes: VOCs, volatile organic compounds; n.s., not specified.

## 4. Killer Toxins: From a Competitive Advantage to the Application as Bio-Drugs

The killer yeast phenomenon is raising interest due to the broad spectrum of activity against human and animal fungal and bacterial infections of yeast killer toxins and due to the recent identification of genes involved in antibiotic resistance and the lack of new antifungal agents [151,152,153,154,155]. The investigated yeast strains and their mechanism of action against human and animal pathogens are summarized in Table 3. *W. anomalus* toxins showed antifungal activity against mouth, bladder and skin *Candida* spp. isolates, as toxins from this species hydrolyze β-1,3-glucans, which are the essential cell wall components of most fungal cells [156]. *Zygosaccharomyces bailii zygocin was active against Candida albicans, C. glabrata, C. krusei and Sporothrix schenckii* [157]. *Pichia kudriavzevii* RY55 toxin exhibited excellent antibacterial activity against several pathogens of human health significance, such as *E. coli, Enterococcus faecalis, Klebsiella* sp., *Staphylococcus aureus*, *Pseudomonas aeruginosa* and *Pseudomonas alcaligenes* [158].

However, the direct application of killer toxin is limited due to the glycoproteic nature of mycocins that may lead to immune response in human bloodstream due to antigenicity and toxicity [159]. Moreover, the activity range of mycocins is restricted, as temperature and pH are limiting factors for most of the killer toxins, which are inactive over 37 °C and at neutral pH. The reason for such behavior is possibly due to the source of isolation, usually fermented food or environmental samples, as recent studies from Cappelli *et al*. [160] reported a killer toxin strain, *W. anomalus* WaF17.12, isolated from a different source, the malaria vector *Anopheles stephensi* mosquitoes, which is active in a wider range of pH (4.5–8.0).

**Table 3:** Medical and veterinary applications of the most studied killer yeast strains and the mechanisms of action.

Species	Yeast Strain	Mechanism	Application	References
*W. anomalus*	WaF17.12	β-1,3-glucanase	In *Anopheles stephensi* agst. *Plasmodium* (Malaria)	[160]
*W. anomalus*	ATCC 96603	β-1,3-glucanase	Antibiobodies agst. *Candida*	[161,162]
*W. anomalus*	ATCC 96603	β-1,3-glucanase	Single-chain fragments (scFv) agst. *Candida* spp., *Staphylococcus* spp., *Enterococcus* spp., oral *Streptococcus*, *Cryptococcus neoformans*	[163,164]
*W. anomalus*	ATCC 96603	β-1,3-glucanase	scFv (single chain Fragment variable) agst. *Paracoccidioides brasiliensis* and *Malassezia pachydermatis* in animals	[165,166]
*W. saturnus* var*. mrakii*	IFO 0895	Inhibition of cell wall β- 1,3-glucan synthase	Antibiobodies agst. *Candida* and *Cryptococcus*	[167,168]

Based on the anti-idiotypic network theory, studies from Polonelli and Morace [161], starting from the identification of biologically-active fragments from *W. anomalus* killer toxin (formerly *Pichia anomala*; PaKT), lead to the production of monoclonal antibodies able to neutralize the activity of PaKT (mAbKT4). Such a finding allowed the production of anti-idiotypic antibodies (anti-id abs) that competed with PaKT for the binding site of mAbKT4 and that were active against *C. albicans*. The so-called “antibiobodies” (antibiotic-like antibodies) showed direct fungicidal effects without the intervention of other factors [162]. Through the immunization of rats and mice against mAbKT4, other monoclonal antibiobodies were obtained, as well as peptides that occur during the idiotypic cascade (mAb K10 and mAb K20) and single-chain fragments (scFv) [163]. In particular, from scFv H6.

Optimized by alanine scanning, a synthetic peptide called KP (AKVTMTCSAS) that demonstrated a wide *in vitro*, *in vivo* and *in planta* antimicrobial activity was obtained and tested against a wide spectrum of pathogens, such as *C. albicans*, *Pneumocystis carinii*, *Mycobacterium tuberculosis*, *S.* *aureus*, *S. haemolyticus*, *E. faecalis*, *E. faecium* and *Streptococcus pneumoniae*. Killer decaPeptide (KP) exerted a strong fungicidal activity, not only against *C.* *albicans*, but also against capsular and acapsular *Cryptococcus* *neoformans* cells, as β-glucan, which is the KP target, is a critical component of the cryptococcal cell wall [164]. The application of killer antibiobodies and their engineered derivatives demonstrated their potential in the prevention of dental caries due to their activity against oral Streptococci [155]; the KP therapy against paracoccidioidomycosis (PCM), which is endemic among individuals living and working in rural areas, especially in South Africa, was able to markedly reduce the *Paracoccidioides brasiliensis* load in organs (liver, lung, spleen) of infected animals [165]. Lastly, KP was effective against *Malassezia pachydermatis*, both *in vitro* and *in vivo*, reducing clinical symptoms and population size of *M. pachydermatis* in the ear canal of dogs affected by otitis [166].

Similar studies were conducted by Selvakumar *et al.* and Kabir *et al.* [167,168], who constructed an anti-idiotypic scFv phage library of *W. saturnus* var*. mrakii* IFO 0895 HM-1 killer toxin using the splenic lymphocytes of mice immunized by idiotypic vaccination with the HM-1 killer toxin neutralizing monoclonal antibody (nmAb-KT). The mechanism of cytocidal activity of HM-1 and scFv antibodies was the inhibition of cell wall β-1,3-glucan synthase, a trans-membrane enzyme responsible for synthesizing the cell wall component β-1,3-glucan [169,170]. Kabir *et al.* [161] published a study on the antifungal potential of peptides derived from both anti-idiotypic antibody and its original fungicidal protein; in particular, SP3 or SP6 peptides proved their potential against *Candida* and *Cryptococcus* species infections and as a promising adjunct for conventional antibiotics. Furthermore, *S. cerevisiae* K2 toxin, with a C-terminal truncation, was obtained in *E. coli* as the host for large-scale production and suitable for polyclonal antibody production [171].

In light of the strong need for new antimicrobial drugs, killer toxin antibiobodies and derived peptides thereof, which can be easily produced and engineered, are emerging as an important class of therapeutic agents for the treatment of various human diseases [172].

## 5. Conclusions

Yeast constitute a large group of microorganisms characterized by a strong ability to compete with other microorganisms for niche colonization. The competition mechanisms have been extensively studied, and among them, killer toxins seem to play a primary role. Killer yeast species have a large biodiversity, in terms of molecular characteristic, genetic determinants, spectra of action and mechanisms of toxin action. Nevertheless, only a small fraction of recognized killer toxins has been characterized in detail so far.

The possibility that additional unknown toxic mechanism in other killer yeast species may occur, together with the potential of known killer toxins to be applied in the food industry as adjunct bioprotective cultures or as a component of active packaging, represents a promising strategy to reduce the use of chemical preservatives. Moreover, the development of killer toxins into a new generation of antimicrobial agents with useful application in the pharmaceutical and medical sectors, for the treatment of microbial infections with resistance to conventional drugs, should represent in the coming future a further boost to the research on this topic.
